# PERK Overexpression-Mediated Nrf2/HO-1 Pathway Alleviates Hypoxia/Reoxygenation-Induced Injury in Neonatal Murine Cardiomyocytes via Improving Endoplasmic Reticulum Stress

**DOI:** 10.1155/2020/6458060

**Published:** 2020-03-26

**Authors:** Jichun Wang, Li Lu, Sisi Chen, Jing Xie, Shuai Lu, Yanli Zhou, Hong Jiang

**Affiliations:** ^1^Department of Cardiology, Renmin Hospital of Wuhan University, Wuhan 430060, Hubei, China; ^2^Cardiovascular Research Institute, Wuhan University, Wuhan 430060, Hubei, China; ^3^Hubei Key Laboratory of Cardiology, Wuhan 430060, Hubei, China

## Abstract

Reperfusion processes following acute myocardial infarction (AMI) have been reported to induce additional cardiomyocyte death, known as ischemia-reperfusion (I/R) injury. Endoplasmic reticulum (ER) stress is reported to be involved in the development of I/R injury. There is evidence that PERK exerts beneficial roles in alleviating ER stress. Here, we investigated whether upregulation of PERK improved cardiomyocytes injury induced by I/R. Specific siRNAs or adenovirus vectors were incubated with isolated neonatal cardiomyocytes (NCMs) to regulate expression levels of target genes including PERK, Nrf2, and HO-1. Afterwards, hypoxia and subsequent reoxygenation (H/R) administration was performed as the in vitro model of I/R injury. MTT assay showed that H/R intervention decreased the viability of cells, yet PERK overexpression increased the cellular proliferative rate. Moreover, the upregulation of Nrf2 or HO-1 elevated the growth rate of cells, while gene silencing of Nrf2 or HO-1 reduced the viability of NCMs treated with PERK-rAAV9. In addition, we observed that the apoptotic index of cells with H/R stimulation was reduced when NCMs were pretreated with PERK-rAAV9, Nrf2-rAAV9, or HO-1-rAAV9. After cells were incubated with Nrf2-siRNA or HO-1-siRNA, the upregulation of PERK had no roles in affecting the apoptosis rate of NCMs damaged by H/R. Then, our findings indicated that there was a level decrease of GRP78, CRT, CHOP, and Caspase-12 in NCMs of the PERK-rAAV9 group compared to that of the H/R group. Both Nrf2 overexpression and HO-1 upregulation reduced the expression of ER stress-related proapoptotic factors, yet the expression suppression of Nrf2 and HO-1 increased levels of GRP78, CRT, CHOP, and Caspase-12 in NCMs treated with PERK-rAAV9. Taken together, our results suggested that the effects of PERK against H/R injury might be attributed to the upregulation of Nrf2/HO-1 cascade, followed by the inhibition of ER stress-related apoptotic pathway.

## 1. Introduction

Epidemiological data has established that acute myocardial infarction (AMI) remains a major cause of death in the world. Due to the stenosis or occlusion of the coronary artery, there is a lack of blood and oxygen supply in distal myocardium tissues, resulting in myocardial ischemic necrosis [1]. As the dead cardiac myocytes lose the ability to contract, the lethal cardiac shock might occur at the acute phase. Additionally, viable myocardial cells are unable to regenerate to repair the infarcted myocardium, and the cardiac systolic function decreases, finally leading to the occurrence of heart failure [2]. Thus, early reperfusion strategies including intravascular thrombolysis and stent angioplasty are of vital importance to limit infarct size and improve the prognosis of AMI patients [3]. However, there is evidence that the reperfusion process per se following the coronary revascularization could induce additional myocardial damage [4, 5].

Ischemia/reperfusion (I/R) injury refers to a pathological phenomenon that reintroduction of oxygen and other nutrients into the myocardium previously supplied by the occluded artery results in additional cell death that is above and beyond the original MI area [6]. Laboratory data has identified multiple potential mechanisms implicated in the detrimental effects of I/R damage, including inflammatory response, the excess formation of reactive oxygen species (ROS), intracellular calcium overload, and rapid normalization of pH [7, 8]. Nonetheless, strategies targeting these pathogenic factors for improving myocardial I/R injury are limited, and the effects of these approaches in the clinical setting have been unsatisfactory until now [9].

The endoplasmic reticulum (ER) is a dynamic cellular organelle that participates in various cellular activities. When the biological functions of ER are disrupted by pathological stimuli, excessive unfolded and misfolded proteins accumulate in the ER to trigger evolutionarily conserved signal-transduction events and then perturb energy and nutrient homeostasis, also called ER stress. Mounting evidence has demonstrated that ER stress is implicated in the development of myocardial I/R damage. There is an increase in the levels of glucose-regulated protein 78 kDa (GRP78), X-box binding protein 1, and eukaryotic translation initiation factor 2*α* of cardiac tissues suffering from I/R injury. After the administration with hypoxia/reoxygenation (H/R), cardiomyocytes have elevated expressions of C/EBP homologous protein (CHOP), protein kinase-like ER kinase (PERK), and calreticulin (CRT) [10–12]. Moreover, the protective actions of several pharmacological interventions against myocardial I/R injury are ascribed to the suppression of ER stress-related activities, such as the expression inhibition of activating transcription factor 6, CHOP, and GRP78 [13]. Interestingly, there is evidence indicating that the upregulation of PERK is capable of alleviating ER stress-evoked cellular death, whereas the molecular mechanisms are poorly understood [14]. Thus, in this study, the hypoxia and subsequent reoxygenation administration of neonatal murine cardiomyocytes was used as an in vitro model of I/R injury to assess whether the upregulation of PERK improved H/R-induced cardiomyocytes death and explore relevant signaling pathways.

## 2. Methods and Materials

### 2.1. Cell Harvest and Culture

Neonatal Kunming mice (1 to 3 days old) were offered by the Hubei Center for Disease Control and Prevention (Wuhan, China). All animal procedures complied with the National Institutes of Health Guide for the Care and Use of Laboratory Animals. This study was carried out with the approval of the Animal Care and Use Committee of the Renmin Hospital of Wuhan University.

The isolation of neonatal cardiomyocytes (NCMs) of Kunming mice was performed in accordance with the enzymatic dissociation method as previously reported. Briefly, the mice were disinfected with 75% ethanol and euthanized by decapitation. The heart was rapidly removed and the ventricular was cut into approximately 1 mm^3^ tissue blocks. Then, the dissected tissues were minced in ice-cold PBS and digested with 0.125% trypsin solution (Gibco, NY, USA). The digestion was terminated by adding fetal bovine serum (FBS) at a final concentration of 20%. Then, the supernatant was gathered and centrifuged at 300 g for 5 min, and the cells were suspended in Dulbecco's modified Eagle's medium: Nutrient Mixture F-12 (DMEM/F12, Gibco, NY, USA) supplemented with 10% FBS and 1% penicillin/streptomycin. The cells were plated into 100 mm culture dishes and incubated for 60 min at 37°C with 5% CO_2_. Then, adherent noncardiac myocytes were discarded and the suspended cells were plated into six-well plates at 37°C for 24 h. Afterwards, 5-bromo-2′-deoxyuridine (Sigma, MO, USA) was added to the culture medium for 48 h to inhibit the proliferation of fibroblasts. The isolated NCMs were then maintained in fresh DMEM/F12 for further study. Primary antibodies against cardiac troponin I and *α*-sarcomeric actin (Abcam, UK) were used for immunofluorescence staining.

### 2.2. siRNA Transfection

For the knockdown experiment of target genes, obtained NCMs were treated with serum-free DMEM/F12 for 24 h. Then, the cells were transfected with PERK-siRNA, Nrf2-siRNA, or HO-1-siRNA using Opti-MEM (Invitrogen, USA) and Lipofectamine 2000 transfection reagent (Thermo Fisher, USA) according to the manufacturer's protocols. The siRNA and Lipofectamine 2000 were separately diluted by Opti-MEM serum-free medium for 5 min. Then, the above two mixtures were blended and kept in room temperature for 20 min. The combined solution was then coincubated with NCMs in the culture plates. After administration for 6 h at 37°C, the mixed medium was discarded and DMEM/F12 containing 10% FBS was added to the plates for the next step. The siRNA used here was as follows: mus PERK-siRNA: 5′-CCUUGGUUUCAUCUAGCCUTT-3′, mus Nrf2-siRNA: 5′-CGAGAAG UGUUUGACUUUATT-3′, mus HO-1-siRNA: 5′-GCCACACAGCACUAUGUAATT-3′, normal control siRNA: 5′-UUGUCCGAACGUGUCACGUTT-3′. The knockdown capacity of siRNA transfection was determined by real-time RT-PCR. The primers were listed in Table 1.

### 2.3. Infection of Adenovirus in NCMs

NCMs at 70%–80% confluence were infected with adenovirus to overexpress target genes including PERK, nuclear factor erythroid 2-related factor 2 (Nrf2), and heme oxygenase-1 (HO-1). The recombinant adeno-associated virus of type-9 (rAAV9) vector delivery system was applied to achieve the target gene-specific upregulation in vitro. A multiplicity of infection was used for the rAAV9 to induce the overexpression of target genes in the NCMs. Then, the fresh culture medium was added to the plates, and then the cells were used for further experiments. Real-time RT-PCR was used to assess the overexpression of target genes in NCMs.

### 2.4. Hypoxia-Reoxygenation Procedure

The hypoxia and subsequent reoxygenation processes were performed as the model of I/R in vitro. Following the infection with rAAV9 or siRNA, NCMs were subjected to H/R administration. Briefly, the cells were exposed to glucose-free and serum-free culture medium and placed in a hypoxia chamber filled with a premixed gas (1% O_2_, 5% CO_2_, and 94% N_2_) for 60 min at 37°C. Afterwards, NCMs were treated with fresh DMEM/F12 containing 10% FBS and transferred into an incubator chamber for 24 h under the normoxic condition (5% CO_2_ and 95% air). Then, the cells and relevant supernatants were harvested for the next step. All NCMs were divided into the following groups according to different interventions: control, H/R, PERK-rAAV9 plus H/R, PERK-siRNA plus H/R, Nrf2-rAAV9 plus H/R, HO-1-rAAV9 plus H/R, Nrf2-siRNA followed by PERK-rAAV9 plus H/R, HO-1-siRNA followed by PERK-rAAV9 plus H/R, and HO-1-siRNA followed by Nrf2-rAAV9 plus H/R. The cells in the control group were continuously cultured in complete DMEM/F12 containing 10% FBS in a regular incubator (5% CO_2_ and 95% air).

### 2.5. MTT Assay

The cell viability was measured via an MTT assay in accordance with the manufacturer's instructions (Sigma, MO, USA). Briefly, isolated NCMs were seeded into 96-well plates at a density of 1.0 × 10^4^ cells/well. After overnight culture at 37°C under 5% CO_2_, the cells were treated with interventions as above described. Then, 10 *μ*L of MTT reagent was added to each well, accompanied by incubation at 37°C for 4 h. The MTT solution was discarded and 150 *μ*L of dimethyl sulfoxide was added for 10 min. The absorbance at 570 nm was examined to analyze the cell viability.

### 2.6. Detection of Cell Apoptosis

The apoptosis rate of cells was measured by the terminal deoxynucleotidyl transferase-mediated dUTP nick-end labeling (TUNEL) kit following the manufacturer's protocols (Roche, Mannheim, Germany). The slides were fixed in 4% paraformaldehyde solution for 25 min and subsequently washed three times with PBS. Then, cell slides were immersed in PBS containing 0.1% TritonX-100 for 2 min, followed by rinsing with PBS. After incubation with the TUNEL reaction mixture at 37°C for 60 min, slides were washed with PBS and incubated with a converter-POD reagent for 30 min, accompanied by rinsing three times. Afterwards, the diaminobenzidine working solution was added onto the cell slides. Images were captured using a Nikon microscope and analyzed by Image Pro Plus 6.0 software. NCMs positively stained with TUNEL were regarded as apoptotic cells and the apoptotic index was defined as the ratio of TUNEL-positive cells relative to all NCMs per field.

### 2.7. Measurement of LDH and CK Level

After the H/R process, obtained cellular supernatant was centrifuged for 10 min at 2000 rpm. The upper liquid was used for the quantification of lactate dehydrogenase (LDH) and creatine kinase (CK) via commercially available kits from Nanjing Jiancheng Bioengineering Institute (Nanjing, China).

### 2.8. Real-Time RT-PCR

Trizol reagent was applied to isolate the total RNA from NCMs according to the manufacturer's instructions (Takara, Japan). A total of 1 *μ*g extracted RNA was used for cDNA synthesis via a reverse transcription kit (Vazyme, China). Then, real-time PCR was performed using SYBR Green Master Mix (Vazyme, China) with an ABI QuantStudio 6 real-time PCR System (Thermo Fisher, USA). The primers used in this study were listed in Table 1. Relative expression levels of mRNAs were calculated by the 2^−△△Ct^ method. GAPDH was considered as the internal control of target genes.

### 2.9. Statistical Analysis

Data in this study was represented as the mean ± standard deviation (SD). Differences between the groups were analyzed using one-way analysis of variance followed by Student-Newman-Keuls multiple comparison test. All statistical analyses were performed using SPSS 19.0 software. A value of *P* < 0.05 was considered statistically significant.

## 3. Results

### 3.1. Identification of NCMs

**Figure 1:**
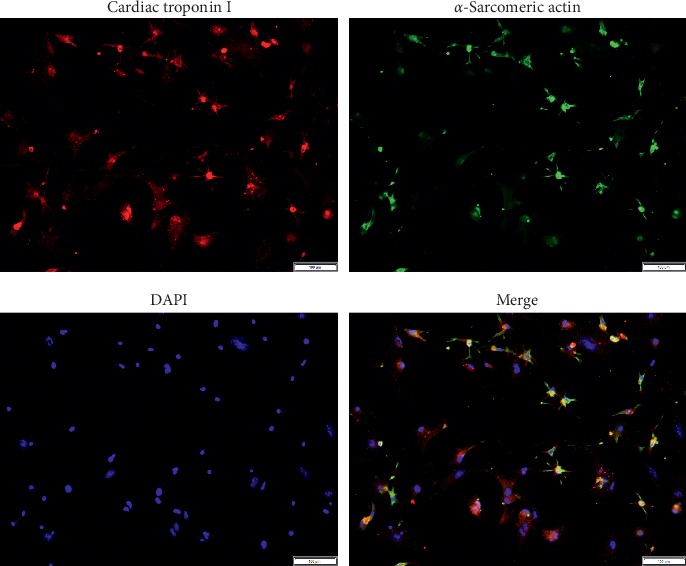
The expression of cardiac troponin I and *α*-sarcomeric actin in the isolated cells was detected by immunofluorescence staining.

In order to ascertain whether the cultured cells isolated from the mice heart tissues were myocardial cells, we detected the expression of cardiomyocyte-related specific biomarkers including cardiac troponin I and *α*-sarcomeric actin in this study. As shown in Figure 1, cardiac troponin I and *α*-sarcomeric actin were both expressed in the extracted karyocytes, verifying the myocardial features of the isolated cells.

### 3.2. The Effects of siRNA and rAAV9 Vector on Gene Expression

**Figure 2:**
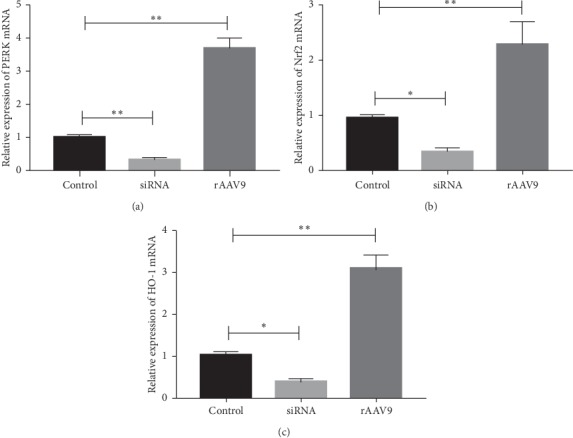
The mRNA expression contents of PERK, Nrf2, and HO-1 in NCMs with different interventions. The mRNA level was normalized to GAPDH. Data was shown as mean ± SD. ^*∗*^*P* < 0.05, ^*∗∗*^*P* < 0.01.

After the incubation of NCMs with specific siRNA or rAAV9, we evaluated the expression levels of target genes. Results of real-time PCR (Figure 2) showed that siRNA treatment and rAAV9 administration significantly triggered the increase and reduction of mRNAs levels, respectively, including PERK, Nrf2, and HO-1 mRNA. These data indicated the high efficiency of siRNA and rAAV9 intervention in regulating the expression of target genes in this study.

### 3.3. PERK/Nrf2/HO-1 Pathway Affected NCMs Viability under the Stimulation of H/R

**Figure 3:**
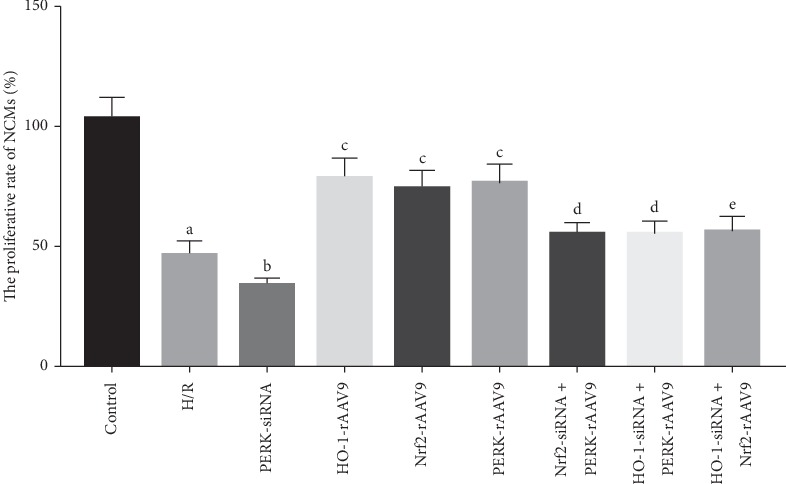
The cellular proliferative rate was measured by MTT assay. NCMs were divided into the control group and the H/R group. Then, cells in the H/R group were further separated into the following groups: H/R intervention alone, PERK-siRNA transfection followed by H/R administration, HO-1-rAAV9 infection accompanied by H/R intervention, Nrf2-rAAV9 treatment accompanied by H/R intervention, PERK-rAAV9 infection followed by H/R administration, Nrf2-siRNA transfection accompanied by PERK-rAAV9 treatment and then H/R intervention, HO-1-siRNA transfection accompanied by PERK-rAAV9 treatment and then H/R intervention, and HO-1-siRNA treatment followed by PERK-rAAV9 infection and then H/R administration. Data was shown as mean ± SD. (a) *P* < 0.01 vs control group; (b) *P* < 0.05 vs H/R group; (c) *P* < 0.01 vs H/R group; (d) *P* < 0.05 vs PERK-rAAV9 group; (e) *P* < 0.05 vs Nrf2-rAAV9 group.

Considering that the H/R process played detrimental roles in cellular viability, the MTT assay was performed (Figure 3). Our findings manifested that the proliferative rate of NCMs was decreased after H/R administration. Meanwhile, we found that PERK overexpression alleviated H/R-evoked growth inhibition of cells, and downregulation of PERK aggravated the impairment of NCMs viability induced by H/R. Then, to investigate the protective mechanisms of PERK, we assessed the effects of Nrf2 and HO-1 on cellular viability in the H/R environment, by the fact that the Nrf2/HO-1 cascade had beneficial roles in cell growth and PERK was a crucial upstream regulator mediating Nrf2/HO-1 activation. Our results indicated that the level elevation of the upregulation of both Nrf2 and HO-1 increased the proliferation rate of cells. Moreover, PERK-rAVV9 intervention had failed to mitigate H/R-induced viability reduction of NCMs when they were pretreated with Nrf2-siRNA or HO-1-siRNA. Similarly, the preconditioning of HO-1-siRNA counteracted the protective effects of Nrf2-rAVV9 on cells.

### 3.4. PERK/Nrf2/HO-1 Pathway Improved H/R-Induced NCMs Damage

**Figure 4:**
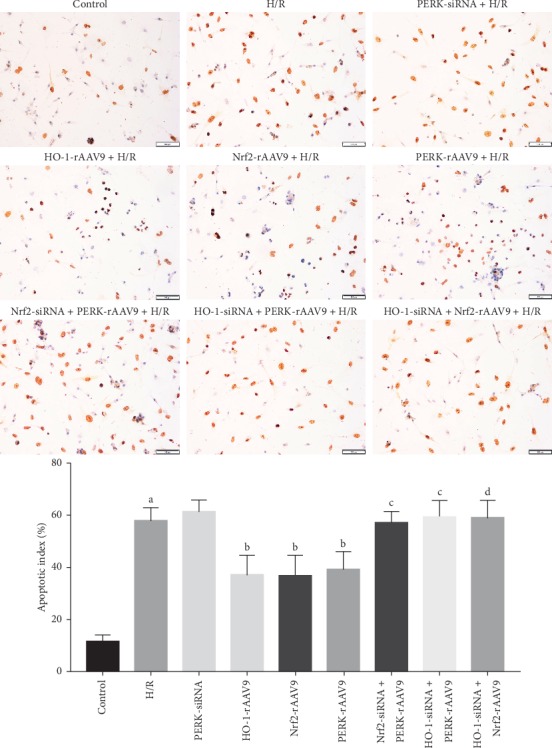
The apoptosis of NCMs induced by H/R was evaluated by TUNEL staining. The apoptotic NCMs referred to the TUNEL-positive cells (brown). The apoptotic index was defined as the ratio of TUNEL-positive cells relative to all NCMs per field. Data was shown as mean ± SD. (a) *P* < 0.01 vs control group; (b) *P* < 0.05 vs H/R group; (c) *P* < 0.05 vs PERK-rAAV9 group; (d) *P* < 0.01 vs Nrf2-rAAV9 group.

For further exploring whether PERK was involved in NCMs injury provoked by H/R, the TUNEL staining and contents of biomarkers reflecting myocardial damage were evaluated. As shown in Figure 4, an increase in the amount of TUNEL-positive cells was seen in the H/R group when compared with that in the control group. The PERK-rAAV9 intervention lowered the apoptosis index of NCMs stimulated by H/R. Furthermore, as Nrf2 and HO-1 were deeply implicated in activities of cellular survival, the regulatory roles of Nrf2 and HO-1 in H/R-induced NCMs apoptosis were determined. We observed that the expression increase of Nrf2 and HO-1 effectively reduced the apoptosis index of NCMs. In addition, gene silencing of Nrf2 and HO-1 dramatically weakened the improvement of cellular survival induced by PERK overexpression. Then, we found that the level elevation of Nrf2 was unable to alter the apoptosis rate of NCMs damaged by the H/R process when cells were pretreated with HO-1-siRNA.

Similarly, the concentrations of CK and LDH were elevated in response to H/R administration (Table 2). After infection with PERK-, Nrf2-, or HO-1-rAAV9, NCMs were found to release decreased contents of CK and LDH as compared to that upon I/R injury alone. Moreover, HO-1-siRNA intervention directly increased CK and LDH release of cells which overexpressed PERK or Nrf2. Taken together, these findings demonstrated that the upregulation of PERK was capable of improving I/R-induced NCMs injury via mediating the downstream Nrf2/HO-1 pathway.

### 3.5. PERK-Regulated Nrf2/HO-1 Cascade Ameliorated H/R Injury via Inhibiting ER Stress

**Figure 5:**
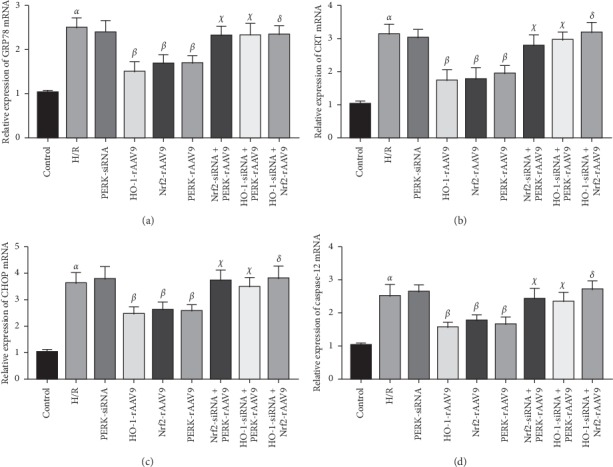
The mRNA expression of ER stress-related proapoptotic molecules in NCMs with different interventions. Data was shown as mean ± SD. ^*α*^*P* < 0.05 vs control group; ^*β*^*P* < 0.05 vs H/R group; ^*χ*^*P* < 0.05 vs PERK-rAAV9 group; ^*δ*^*P* < 0.05 vs Nrf2-rAAV9 group.

Then, we further investigated the molecular mechanisms underlying the fact that PERK-mediated Nrf2/HO-1 axis alleviated cell damage triggered by H/R process. Owing to the fact that ER stress-related signaling factors were involved in the development of reperfusion injury, we discovered that expressions of GRP78 and CRT were elevated in the H/R group compared to those in the control group. PERK overexpression effectively reduced the contents of GRP78 and CRT in NCMs upon H/R stimulation. In addition, our results showed that both Nrf2-rAAV9 and HO-1-rAVV9 lowered the expressions of GRP78 and CRT. After NCMs were pretreated with Nrf2-siRNA or HO-1-siRNA, the upregulation of PERK was incapable of reducing the expression levels of GRP78 and CRT. NCMs in HO-1-siRNA transfection followed by the Nrf2-rAVV9 infection group had higher levels of the ER stress-related molecules than those in the Nrf2-rAVV9 infection alone group. Moreover, we found that CHOP and Caspase-12, which possessed pivotal roles in facilitating ER stress-induced cell death, exhibited the same tendency of expression change as GRP78 and CRT in this study (Figure 5). Thus, our data suggested that the inhibition of ER stress-related signal molecules and the downstream cell death pathway might be pivotal contributors to the protective effects of PERK-mediated Nrf2/HO-1 axis against H/R injury.

## 4. Discussion

Although the reperfusion strategies including thrombolytic therapy and angioplasty have been widely applied, AMI remains a leading cause of death and disability worldwide [15]. An important reason for this phenomenon is that the I/R process following AMI induces additional cardiomyocyte death and then extends the MI area, thereby deteriorating the prognosis of patients [4, 5, 16]. In this study, we used hypoxia and subsequent reoxygenation administration of NCMs as a model of I/R injury and found that H/R process markedly triggered NCMs damage. Yet, the upregulation of PERK and downstream Nrf2/HO-1 pathway effectively improved cellular injury induced by H/R, the molecular mechanism for which might be attributed to the expression inhibition of ER stress-related factors and relevant proapoptotic signal molecules.

It has been established that, during the process of I/R, cellular death-related pathways are activated, leading to mitochondrial dysfunction, apoptotic protein leakage, cytomembrane disruption, and myocardial enzyme release [7, 17]. In line with previous experimental data, our findings indicated that there were impaired characteristics exhibited in NCMs after the administration of H/R, as evidenced by proliferation rate reduction, number increase of TUNEL-positive cells, and contents elevation of CK and LDH. PERK, acting as a crucial signaling molecule associated with the development of ER stress, has been reported to inhibit the accumulation of unfolded proteins in the ER and then regulate ER stress in a negative feedback way [14]. Recent studies have provided evidences that several therapies capable of activating PERK cascade exert beneficial roles in improving cellular survival rate. Zhang et al. clarify that the upregulation of PERK signaling alleviates apoptosis of skeletal muscle cells under high-stress conditions of hibernation [18]. In addition, data from other studies manifests that the silencing of PERK contributes to the apoptosis of chondrocytes and pancreatic *β*-cells [19, 20]. Our results suggested that PERK overexpression markedly inhibited H/R-induced NCMs apoptosis, which demonstrated the cellular protective roles of PERK.

Nrf2, known as a nuclear transcriptional factor, has been demonstrated to possess pleiotropic functions in maintaining cellular homeostasis via regulating the expression of downstream effectors, such as HO-1 [21, 22]. Several lines in the literature indicate that PERK-activated Nrf2 signaling transduction displays pivotal roles in cellular protection. For instance, Yang et al. report that Tang-Luo-Ning treatment obviously mitigates hyperglycemia-provoked Schwann cells death via upregulating PERK/Nrf2 cascade [23]. The mechanism underlying the fact that Botrysphin D relieves the impairment of lung epithelial cells caused by arsenic is attributed to the activation of the PERK-mediated Nrf2 pathway [24]. However, other studies report that Nrf2 upregulation might induce mitochondrial injury and ROS overproduction, resulting in cardiomyocytes apoptosis. [25]. Here, we investigated whether PERK ameliorated H/R-induced cardiomyocyte death by modulating the Nrf2/HO-1 signal axis. Our findings demonstrated that all of the PERK-, Nrf2-, and HO-1-rAAV9 groups had higher cell growth rate and lower apoptotic index as compared to the H/R group, yet there were no differences in the damage-related parameters among the three groups. Moreover, we discovered that cellular protective roles of PERK overexpression were invalid when NCMs were pretreated with Nrf2-or HO-1-siRNA, and silencing of HO-1 counteracted the beneficial effects of PERK and Nrf2-rAAV9 on cellular survival. These above results suggested that the inner mechanism by which PERK upregulation improved H/R-triggered cardiomyocytes injury was attributed to the activation of the Nrf2/HO-1 signal pathway.

A growing body of evidence has elucidated that ER stress takes part in the pathogenic processes of I/R injury [26]. Laboratory studies report that GRP78 and CRT, serving as key signaling molecules involved in ER stress-related pathways, are highly expressed in the tissues which suffer from I/R damage [27–29]. Moreover, in vitro experiments show that there exist increased levels of GRP78 and CRT in the cells after administration with H/R [30, 31]. Consistent with previous data, our findings indicated that the expression contents of GRP78 and CRT were increased in the H/R group and the upregulation of PERK/Nrf2/HO-1 cascade obviously reduced levels of GRP78 and CRT, suggesting that PERK-activated Nrf2/HO-1 pathway was capable of alleviating ER stress during the H/R process. It has been verified that CHOP is responsible for the regulation of ER stress-mediated cellular apoptosis [32, 33]. When the upstream signal stimulation persists, CHOP expression is elevated, followed by an increase of Caspase-12, ultimately leading to the initiation of apoptotic activities. Several studies demonstrate that suppression of CHOP/Caspase-12 signal flow is beneficial for alleviating ER stress-induced cell apoptosis and then improving H/R injury [34–36]. Due to the vital roles of CHOP/Caspase-12 cascade in H/R injury and inhibitory effects of the PERK/Nrf2/HO-1 axis on ER stress development, we postulated that the overexpression of the PERK and downstream Nrf2/HO-1 pathway possibly improved H/R damage through reducing ER stress-related proapoptotic signal factors. As expected, we found that the upregulation of PERK/Nrf2/HO-1 significantly decreased the levels of CHOP and Caspase-12, and gene silencing of Nrf2 or HO-1 abrogated the inhibitory roles of PERK in the expression of CHOP/Caspase-3. These above results indicated that the PERK-activated Nrf2/HO-1 axis might attenuate NCMs injury induced by H/R via suppressing ER stress signals, accompanied by restraint of CHOP/Caspase-12 pathway and blockade of downstream apoptotic signaling transduction.

However, there were several limitations to this study. Firstly, the potential mechanisms underlying the fact that Nrf2/HO-1 inhibited ER stress-related pathways were poorly understood, which remained to be elucidated. In addition, owing to the fact that there were multiple cell types included in myocardial tissues, such as cardiomyocytes, fibroblasts, vascular endothelial cells, and lymphocytes, the effects of PERK overexpression on the development of I/R injury of these cells except for cardiomyocytes were needed to be investigated. Moreover, given that in vivo experiments were essential for assessing the precise effects of specific strategies, the study procedures should be transferred into the animal model for further evaluating the therapeutic value of PERK/Nrf2/HO-1 cascade against I/R injury.

In summary, the present study suggests that the increased expression of PERK displays beneficial roles in improving H/R-induced NCMs injury. The cellular protective effects of PERK might be attributed to the upregulation of the Nrf2/HO-1 cascade, accompanied by inhibition of ER stress, thereby suppressing the activation of the downstream apoptotic pathway. This study provides novel insights into the therapeutic potential of PERK as a valuable target for the management of I/R injury following AMI.

## Figures and Tables

**Table 1:** The primer sequences used for real-time RT-PCR.

Primer	Forward sequence (5′-3′)	Reverse sequence (5′-3′)	Produce size (bp)
PERK	CGGCAGGTCCTTGGTAATCA	GAGGAAGTTTTGTGGGTGCC	156
Nrf2	CAGTGCTCCTATGCGTGAA	GCGGCTTGAATGTTTGTCT	109
HO-1	TTCAGAAGGGTCAGGTGTCC	CAGTGAGGCCCATACCAGAA	193
GRP78	CCATCCCGTGGCATAAAC	TGTCTTTTGTTAGGGGTCGTT	277
CRT	CTGGTCCTTCTTCACCCCAT	TCTGCCATGGTTCCTTTTGC	201
CHOP	TCACTACTCTTGACCCTGCG	ACTGACCACTCTGTTTCCGT	174
Caspase-12	ATTCCTGGTGTTTATGTCCC	TCCATTATATCTGCCTCTGC	184
GAPDH	ATGGGTGTGAACCACGAGA	CAGGGATGATGTTCTGGGCA	229

**Table 2:** The contents of CK and LDH in different groups.

	Control	H/R	PERK-siRNA	HO-1-rAAV9	Nrf2-rAAV9	PERK-rAAV9	Nrf2-siRNA + PERK-rAAV9	HO-1-siRNA + PERK-rAAV9	HO-1-siRNA + Nrf2-rAAV9
CK (U/L)	326.91 ± 33.23	734.82 ± 59.11^a^	714.76 ± 81.55	505.35 ± 79^b^	539.12 ± 61.49^b^	529.33 ± 55.69^b^	694.35 ± 92.25^c^	685.88 ± 87.54^c^	697.64 ± 98.57^d^
LDH (U/L)	120.93 ± 15.41	389.45 ± 41.67^a^	378.72 ± 58.02	286.66 ± 36.03^b^	251.49 ± 40.62^b^	274.58 ± 36.79^b^	375.36 ± 59.23^c^	369.13 ± 67.29^c^	357.55 ± 58.18^d^

^a^ *P* < 0.05 vs control group; ^b^*P* < 0.05 vs H/R group; ^c^*P* < 0.05 vs PERK-rAAV9 group; ^d^*P* < 0.05 vs Nrf2-rAAV9 group.

## Data Availability

The data used to support the findings of this study are available from the corresponding author upon request.
